# Experimental Infection of Captive Red Foxes (*Vulpes vulpes*) with *Mycobacterium bovis*

**DOI:** 10.3390/microorganisms10020380

**Published:** 2022-02-06

**Authors:** Céline Richomme, Sandrine Lesellier, Francisco Javier Salguero, Jacques Laurent Barrat, Jean-Marc Boucher, Jennifer Danaidae Reyes-Reyes, Sylvie Hénault, Krystel De Cruz, Jennifer Tambosco, Lorraine Michelet, Justine Boutet, Rubyat Elahi, Konstantin P. Lyashchenko, Conor O’Halloran, Ana Balseiro, Maria Laura Boschiroli

**Affiliations:** 1 Nancy Laboratory for Rabies and Wildlife, ANSES, 54220 Malzéville, France; celine.richomme@anses.fr (C.R.); sandrine.lesellier@anses.fr (S.L.); jlb@jlbperso.fr (J.L.B.); Jean-Marc.BOUCHER@anses.fr (J.-M.B.); 2 United Kingdom Security Agency, Porton Down, Salisbury SP4 0JQ, UK; Javier.Salguero@phe.gov.uk; 3 Laboratory for Animal Health, Tuberculosis National Reference Laboratory, University Paris-Est, ANSES, 94701 Maisons-Alfort, France; jenny2drr@gmail.com (J.D.R.-R.); Sylvie.henault@anses.fr (S.H.); krystel.decruz@anses.fr (K.D.C.); Jennifer.tambosco@anses.fr (J.T.); Lorraine.michelet@anses.fr (L.M.); 4 IUT Génie Biologique, University of Tours, 37000 Tours, France; justine.boutet@etu.univ-tours.fr; 5 Chembio Diagnostic Systems, Inc., 3661 Horseblock Road, Medford, NY 11763, USA; rubyatelahi@gmail.com (R.E.); kLyashchenko@chembio.com (K.P.L.); 6 Royal (Dick) School of Veterinary Studies, University of Edinburgh, Easter Bush, Midlothian EH25 9RG, UK; 7 Departamento de Sanidad Animal, Facultad de Veterinaria, Universidad de León, 24071 León, Spain; abalm@unileon.es; 8 Departamento de Sanidad Animal, Instituto de Ganadería de Montaña (CSIC-Universidad de León), Finca Marzanas, Grulleros, 24346 León, Spain

**Keywords:** tuberculosis, red fox, *Vulpes vulpes*, pathogenesis, excretion, serology

## Abstract

In Europe, animal tuberculosis (TB) due to *Mycobacterium bovis* involves multi-host communities that include cattle and wildlife species, such as wild boar (*Sus scrofa*), badgers (*Meles meles*) and red deer (*Cervus elaphus*). Red fox (*Vulpes vulpes*) infections have also been recently reported in some TB endemic regions in the Iberian Peninsula and France, with some of the infected animals shedding *M. bovis* in urine and feces. In order to understand the pathogenesis of *M. bovis* infection in foxes and the associated risk of transmission, 12 captive foxes (6 females and 6 males) were inoculated orally with 2 × 10^7^ colony-forming units of a French field isolate of *M. bovis*. Clinical samples (urine, feces and oropharyngeal swabs) were collected every four weeks and tested for molecular diagnosis and bacteriology. Serological responses were measured by IDEXX M. bovis Ab Test and Multi Antigen Print Immunoassay (MAPIA). At a post-mortem examination performed 12 weeks post infection (wpi), tissues were tested for the presence of *M. bovis* and associated gross and microscopic TB-like lesions. *M. bovis* was detected by PCR in bladder swabs of 3 animals at 12 wpi. It was also detected pre-mortem at different time points of the experiment in the oropharyngeal mucus of three individuals and in the feces of nine foxes, with two of them confirmed by bacteriology. All 12 foxes had at least 4 PCR positive samples (out of the 23 tested), and all but 1 fox had at least 1 culture positive sample. The culture negative fox was PCR positive in both retropharyngeal and mesenteric lymph nodes, in line with the results of the other animals. Seroconversion was observed in all foxes except one during the experiment, and in nine at the final time point. No gross visible lesions were found in any animal at the post-mortem examination. The histology showed small granulomas within the lymph nodes, tonsils, liver and lungs from eight animals, with the presence of few acid-fast bacilli. These results confirmed that all orally-infected foxes developed mild TB lesions but they were able to shed mycobacteria in about 75% of cases, 1 month post-infection (9 out 12 foxes). These results show that it is possible to induce typical TB infection experimentally in captive foxes, with measurable *M. bovis* excretion; such an experimental system could be useful for future evaluations of diagnostics and vaccines in this species.

## 1. Introduction

Animal tuberculosis (TB) caused by members of the *Mycobacterium tuberculosis* complex (MTBC), mainly *Mycobacterium bovis* and *M. caprae*, is one of the most important infectious animal diseases in Europe. Its control, if not eradication, is urgent for the farming industry, public health (zoonotic infections) and wildlife conservation [1]. TB involves multi-host communities, including livestock species (cattle, goats, sheep), but also wildlife species, such as wild boar (*Sus scrofa*), red deer (*Cervus elaphus*) and badgers (*Meles meles*), in Europe [1]. It is recognized that TB eradication requires controlling the reservoir(s), defined as epidemiologically connected animal populations, in which the pathogen can be transmitted between the species [2].

Red foxes (*Vulpes vulpes*) are considered as a TB host of minor epidemiological importance. In the early 2000s, only 3% of foxes were found to be infected with *M. bovis* in an endemic area in Great Britain where TB prevalence in cattle and badgers was high [3]. More recently, a higher level of infection (27%) was observed in red foxes in Portugal [4]. The different infection rates observed could be due to sampling differences (sampling size and sites, variations in analyzed tissue) or the diagnostic procedure employed, but also to eco-epidemiological differences, i.e., the sympatric host composition of the system, rate of infection of these different hosts and the way foxes contract infection. In the Portuguese study sites, where ungulates were found to be infected in high rates, and mainly in wild boar, the authors suggested that foxes may acquire the infection through the consumption of infected carcasses. In all settings, very few infected foxes presented gross lesions:1 case in the British study (among 756 foxes analyzed) [3] and another described in southern Spain [5]. Due to the scarcity of gross lesions in these reported cases, the risk of *M. bovis* shedding in fox feces, urine and tracheal mucus was assumed to be low. However, in 2018, *M. bovis* was detected in body fluids from four infected red foxes without visible lesions captured in a highly TB prevalent area in France [6]. After the discovery of grouped cases of TB in foxes in a French TB endemic region, a larger study was implemented in three core areas of the TB endemic Nouvelle-Aquitaine region (southwestern France), where the number of cattle outbreaks had been increasing in the last 10–15 years. This study demonstrated that *M. bovis* prevalence in foxes was reaching 5.0% to 9.2%, as high as those observed in badgers and wild boar in the same areas [7]. None of the infected animals presented gross lesions. All these findings raised the question of the putative role of foxes in local TB epidemiology, particularly for transmitting *M. bovis* to cattle, given the known circulation of the bacteria between cattle, badgers, wild boar, roe deer and red deer [8].

The aim of the present study is to develop for the first time a reproducible experimental TB infection in captive red foxes, to study the pathogenesis and mycobacterial excretion in this species.

## 2. Material and Methods

### 2.1. Ethics Statement

The experiment procedure was approved by the French ethical committee for animal experimentation n°16 and by the French “Ministère de l’Enseignement Supérieur et de la Recherche” on 2 October 2019 (APAFIS#16237-2018072316235926v4).

### 2.2. Animals and Samples Collection

Twelve foxes (*Vulpes vulpes*), identified as A to L, were used in the experiment (see Table 1 in Results). Six were males and six females, and all were adults (from 1 to 7 years old), sourced from approved breeding silver fox farms in Finland (officially TB-free in cattle and in any other animal species). The animals were housed in individual cages with internal enrichment. They were fed daily with dried dog food and had ad libitum access to water. All the foxes were monitored daily for food intake, clinical conditions and production of feces and urine.

Just before challenge (time-point 0, T0), 4 weeks post infection (wpi) (time-point 1, T1), 8 wpi (time-point 2, T2) and 12 wpi (time-point 3, T3), blood, oropharyngeal swabs and urine samples were collected under anesthesia with ketamine + xylazine (Imalgen^®^ 1000: 1 mL/10 kg IM + Rompun^®^ 2%: 1 to 1.5 mL/10 kg). Urine samples were collected at eight wpi from all foxes except foxes F and I. Feces were collected in cages from all foxes except for fox H and foxes A, B, C and D, respectively.

For blood collection, the cephalic vein or the jugular vein were punctured using SST vacutainers (BD^®^, Becton, Dickinson and Company, Franklin Lakes, UK). Serum was isolated by centrifugation of the vacutainers (5000 g-15 min) and frozen at −20 °C until use. On each time-point, feces were collected from the cages and frozen at −80 °C before molecular analysis and bacterial culture.

At 12 wpi, the anaesthetized foxes were euthanized using an intravenous (IV) overdose of sodium pentobarbital (euthasol, Dechra veterinary products SAS, Montigny-le-Bretonneux).

### 2.3. Mycobacterium bovis Strain Used for the Infection

The *M. bovis* strain #119 used for the experimental infection was isolated from a naturally-infected fox (RN5), in a French TB endemic area in the Dordogne department, Nouvelle Aquitaine, as described by Michelet et al. in 2018 [6]. This strain belonged to the most common *M. bovis* genotype (SB0120 MLVA 5 3 5 3 9 4 56) isolated in Dordogne in cattle and wildlife.

The infectious *M. bovis* stock was prepared by growing the original strain isolate (stored at −20 °C) in a two-steps process: first in 1 mL of Middlebrook 7H9 + mycobactine until a stationary phase-optical density (OD) of 1, and then in 20 mL of Middlebrook 7H9 until an exponential phase-OD of 0.6. The infectious stock was controlled for purity by Ziehl–Neelsen staining and stored at +4 °C, including during transport. The concentration was established at 2 × 10^7^ colony-forming units (CFU)/mL. The strain was not frozen in order to avoid a decrease in infectivity.

### 2.4. Infection

On the day of infection, 12 *M. bovis* 1 mL stock vials were incubated at 37 °C for 6 h, and each of them injected in 5 times 200 µL (1 mL in total) inside individual pieces of raw beef (of approximately 65 cm^3^).

The animals were fasted for 48 h before infection, and individual foxes each received a piece of meat containing 2 × 10^7^ colony forming units (CFU) of *M. bovis* suspension.

### 2.5. IDEXX M. bovis Ab Test

The IDEXX M. bovis Ab Test was adapted from the IDEXX ELISA originally developed for cattle (GP527, Idexx, Westbrook, ME, USA), where the secondary antibody reagent was replaced by conjugate CF2 (Réf. MAB10/60, APHA, New Haw, UK) developed for badger IgG, but also cross-reacting with fox IgG. Plates pre-coated with MPB70 and MPB83 proteins were incubated for 1 h at room temperature with 100 µL of duplicated serum samples diluted 1/10 in the diluent provided by the manufacturer. After washing with the buffer provided by the manufacturer, 100 µL of CF2 diluted at 1/4700 in the diluent was added to each well. After 30 min incubation and a new washing step, 3, 3′, 5, 5′ Tétraméthyl Benzidine (TMB) (Réf. TE265835, Thermoscientific, Pierce Biotechnology, Rockford, IL, USA) was added to each well for 15 min followed by 100 µL of sulfuric acid (Réf. 704362, Scientific Laboratories Supplies, Nottingham, UK). The color intensity in each well was measured at 450 nm wavelength with the ELISA plate reader Thermo Multiskan Ascent (Thermofisher, Waltham, MA, USA). In replacement of fox known positive samples, the serum samples of dogs infected with *M. bovis* were used as positive control samples in the test. Non-TB-infected captive foxes were used as negative samples (12 study animals at pre-challenge T0 and 27 additional captive foxes), and to calculate the cut-off point being the higher limit of the 95% confidence interval around the mean (cut-off point value: 0.107).

### 2.6. MAPIA

Serum samples collected at the different time-points were tested for IgM and IgG antibody responses by Multi Antigen Print Immunoassay (MAPIA), as previously described [9], with minor modifications. Briefly, the following eight protein antigens were immobilized onto the nitrocellulose membrane: ESAT6, CFP10, MPB70, MPB83, E6/P10 (ESAT6-CFP10), DID38 (MPB70-MPB83), DID65 (Rv0934-CFP10-MPB70) and bovine tuberculin purified protein derivative (B-PPD). Test strips were incubated with fox sera collected at all time points at 1:40 dilutions for 1 h at room temperature. Antigen-bound IgM and IgG antibodies were detected by strip incubation with alkaline phosphatase conjugated to goat antibodies to canine IgM or IgG, respectively (Novus Biologicals, Centennial, CO, USA), followed by visualization with substrate 5-bromo-4-chloro-3-indoyl-phosphate/nitroblue tetrazolium (Seracare Life Sciences, Milford, MA, USA). A band of greater visual intensity than that obtained with pre-infection serum sample for the same antigen(s) was considered as an antibody positive result.

### 2.7. Collection of Tissues Post-Mortem

At twelve wpi, the foxes were anesthetized, euthanized and a detailed post-mortem exam was performed following a systematic standard procedure. Tissues (Table 2) were collected using separate sterile sets of instruments and tubes for each tissue: tonsils, left and right retro-pharyngeal, parotid and mandibular lymph nodes, salivary glands, lungs (only for histopathology), spleen, liver, mesenteric and hepatic lymph nodes, and pooled thoracic (mediastinal and right and left bronchial) lymph nodes. Some lymph nodes were not collected in some foxes because they were not found (too small) (Table S1). Tracheal medial section, proximal esophagus section, urine or bladder swabs, and feces from the rectum were also collected. Macroscopical lesions were quantified. Tissues were weighed and one part was submitted for culture and polymerase chain reaction (PCR); the remaining part was collected into 10% buffered formalin for histological analysis (Table 2). A urine sample was absent for fox H and feces for foxes A, B, C and D.

### 2.8. Histopathology

Samples were fixed in buffered formalin and processed routinely for embedding into paraffin wax. Four micron sections were cut and stained with hematoxylin and eosin (H&E) and Ziehl–Neelsen stain for the identification of granulomas and acid-fast bacilli (AFB), respectively. Granulomas within tissue sections were scored for their severity or degree of maturation from I to IV, following a similar scoring system, as previously described for cattle and badgers [10,11]. Briefly, those given score I showed small clusters of activated macrophages and lymphocytes; score II showed larger inflammatory cell infiltration with the presence of neutrophils and minimal necrosis; score III showed a larger necrotic core; while score IV showed caseotic necrosis and lesions were frequently coalescing. The number of granulomas for each score was counted in each H&E stained section. Moreover, the Ziehl–Neelsen stain was used to detect AFB and each tissue was scored as 0 = no presence of AFB, 1 = presence of few AFB and 2 = presence of abundant AFB.

### 2.9. Culture

**Figure 1:**
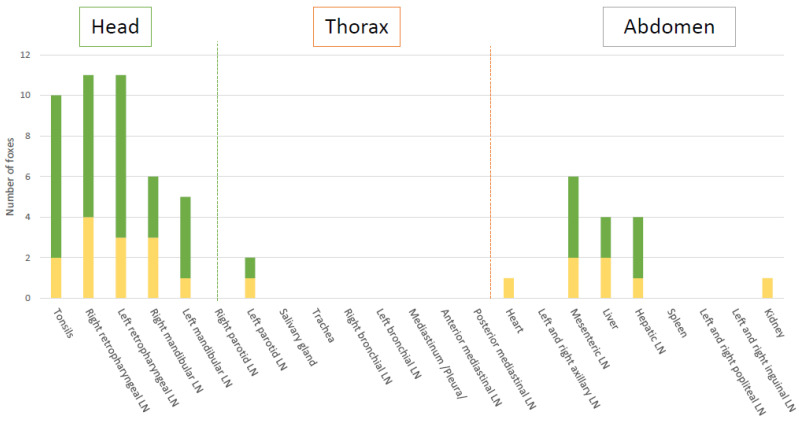
PCR and bacteriology (in green), and PCR only (in yellow) results in all tissues. LN: lymph node.

TB infection was studied by molecular diagnosis and bacterial culture in 23 different tissue samples (see Figure 1 in Results section) and clinical samples.

Tissues were homogenized in sterile tubes (IKA^®^, BMT-20-S, Wilmington, NC, USA) for 1–2 min in 3 mL (or up to 5 mL in bigger samples) of saline solution (Table S1). Fecal samples were decontaminated with 4% NaOH (*w*/*v*) for 15 min at 37 +/− 2 °C, and neutralized with 10% H2SO4 (*v*/*v*). All samples were inoculated onto Modified Middlebrook 7H11 medium (BD DifcoTM Mycobacteria 7H11 Agar, BD Biosciences, Franklin Lakes, NJ, USA).

Washes of tracheal section (~1 cm) in saline, homogenized tissues, oropharyngeal swabs eluted in saline, urine and treated feces were each inoculated onto four Modified Middlebrook 7H11 agar (100 µL per plate) for 12 weeks at 37 °C. *M. bovis* was confirmed by Spoligotyping by Luminex, as described by Zhang et al. [8], using TB-SPOL kits purchased from Beamedex^®^ (Beamedex SAS, Orsay, France) on Bio-PLex 200/Luminex 200^®^ on bacterial colony thermolysates. Live *M. bovis* in tissues was quantified as the number of CFU per gram of tissue before homogenization or CFU in 1.8 mL of homogenized tissue (1.8 mL corresponding to the quantity used for DNA extraction and PCR).

### 2.10. Molecular Diagnosis

After mechanical lysis, DNA was extracted from 1.8 mL of homogenized tissues, urine and tracheal wash using an LSI MagVetTM Universal Isolation Kit (Life Technologies) with a KingFisherTM Flex automate (Thermo Scientific), following the manufacturer’s instructions. Bacterial DNA was extracted from feces with the MP-bio power soil kit [12]. The positive detection of *M. bovis* was established on the basis of a positive response for IS*1081* and/or IS*6110* (*Mycobacterium tuberculosis* complex) [13] by real-time PCR indicated in CT (cycle threshold) values. 

## 3. Results

### 3.1. Clinical Follow Up

No clinical signs were observed during the 12-week study.

### 3.2. Serological Monitoring

The IgG antibody responses to MPB83/MPB70 were positive from 4 wpi (in 10 out of the 12 foxes) by IDEXX. They decreased at 8 wpi (only 5 animals remained positive) and increased again at 12 wpi (9 animals positive). Fox G was strongly positive pre-challenge by Idexx and remained so throughout the experiment.

The responses to a wider range of antigens were detected at 12 wpi by MAPIA, separating IgM and IgG responses (Table 1). All the foxes positive by IDEXX also presented antibody responses detected by MAPIA (6 animals with IgM responses and 5 with IgG responses), whereas 1 animal (Fox J) negative by IDEXX developed an IgM response to B-PPD in MAPIA. Two animals presented both antibody isotypes. If the IgM and IgG results are considered in combination, 9/12 animals produced MAPIA positive results. All seropositive results (except for Fox G) appeared as relatively weak bands suggesting low antibody levels. The IgM and IgG responses were characterized by variable reactivity to five antigens, four of which (E6/P10, DID38, DID65 and B-PPD) were recognized most frequently. Four foxes positive to MPB83-MPB70 by IDEXX also responded by MAPIA, to either the isolated MPB83 antigen or to the combined protein pool DID38 (MPB70-MPB83). Three other foxes (C, E and K), negative by MAPIA to MPB83 or DID38, responded to DID65 (Rv0934-CFP10-MPB70). Four infected foxes responded to ESAT-6-CFP-10. Supplementary Figure S1 shows examples of the IgM and IgG seroconversions observed with various test antigens in different animals. MAPIA results demonstrate that the antibody responses could develop as early as 4 wpi.

### 3.3. Infection of Tissues

**Figure 2:**
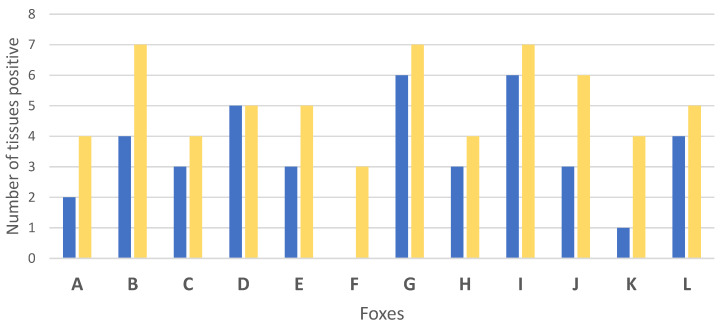
Number of infected tissues by bacteriology (blue) and PCR (yellow) for each of the 12 foxes (A to L) experimentally infected with *Mycobacterium bovis*.

Global results in bacteriology and PCR of the tissues showed that experimentally *M. bovis* infection was successful to induce infection in all challenged foxes (Table 2, Figure 1 and Figure 2). A total of 11 out of the 12 foxes presented live *M. bovis* detected by culture and the remaining culture negative fox was positive by molecular diagnosis in 3 tissues (bilateral retropharyngeal LNs and mesenteric LN) (Figure 1 and Figure 2). Tissue infection ranged between three to eight tissues positive by PCR and bacteriology per fox. Foxes G and I were the most heavily infected according to the bacteriological results and PCR results, with 6 and 7 positive tissues, respectively (Figure 2).

A total of 11 tissue types out the 23 collected were positive by PCR, with 9 of them also positive by bacteriology (Figure 1, Table 2). Of the 262 analyzed samples, 61 resulted positive by PCR. Five tissues of different types had inhibitions, as shown by the Internal Positive Control (IPC), and were still negative for the MTBC PCR after DNA dilution.

**Figure 3:**
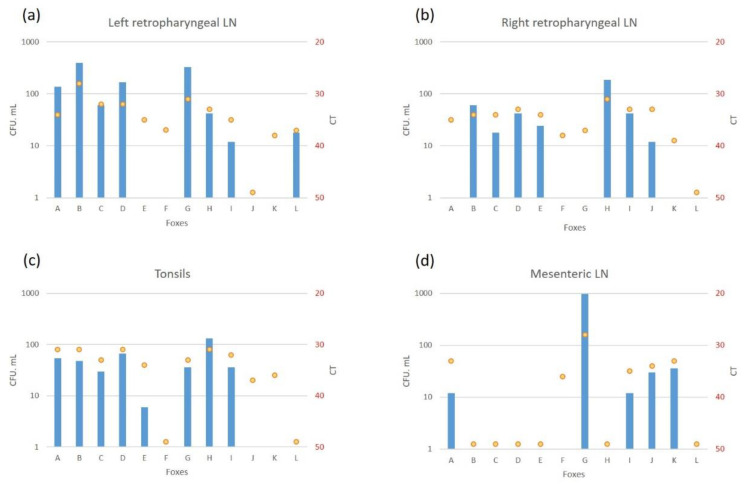
Bacteriology (colony forming units (CFU/1.8 mL) (in blue) and PCR IS*6110* (Cycle threshold (CT)) (yellow points) levels of infection in the left (**a**) and right (**b**) retropharyngeal lymph nodes (LN), tonsils (**c**) and mesenteric LN (**d**).

The retropharyngeal lymph nodes and tonsils were the most frequently infected tissues (8 and 8 foxes positive by culture, and 11 and 10 foxes by PCR, respectively) (Figure 1). The highest bacterial loads observed were in fox B’s retropharyngeal lymph node with 396 CFU/1.8 mL on the left and 60 CFU/1.8 mL on the right (correspondingly 1358 and 264 CFU/g, Table S3). In other foxes, a higher bacteria load was also observed in the left retropharyngeal lymph nodes than in the right ones (Figure 3).

Infection of the thoracic tissues was rare (Figure 1). In the abdomen, the mesenteric lymph node was the most frequently infected tissue detected either by culture or PCR (Figure 1), with bacterial loads between 12 CFU/1.8 mL (163 CFU/g, Table S3) and CT of 35 (fox I) and 966 CFU/1.8 mL (6192 CFU/g, Table S3) and CT of 28 (fox G) (Figure 3). The liver and the hepatic lymph node were positive by PCR in 4 foxes, with 2 of them positive by bacteriology in the liver (D and L), and 3 positive by bacteriology in the hepatic lymph node (G, J and L) (Table S3).

### 3.4. Excretion Monitoring

**Figure 4:**
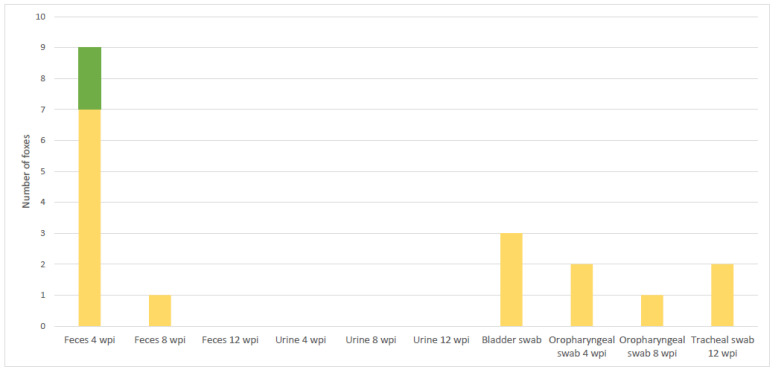
Number of foxes positive by PCR and bacteriology simultaneously (in green), and by PCR only (in yellow) in shedding material.

Urine samples collected per fox at times T1, T2 and T3 were negative either by bacteriology or by PCR in all foxes (Figure 4). Urinary bladder swabs were positive by PCR in 3 foxes at 12 wpi (T3) (Figure 4, Table S4).

All the oropharyngeal swabs were negative by culture. However, 2 swabs were PCR positive at 4 wpi and 1 at 8 wpi, as well as 2 tracheal swabs at post-mortem (Figure 4).

Feces were PCR positive in 9 foxes at 4 wpi, 2 of which (B and C) were also positive by *M. bovis* culture (Figure 4). All other fecal samples were negative (Table S4).

### 3.5. Pathology and Histopathology

No visible lesion was found in any animal at the post-mortem examination.

**Figure 5:**
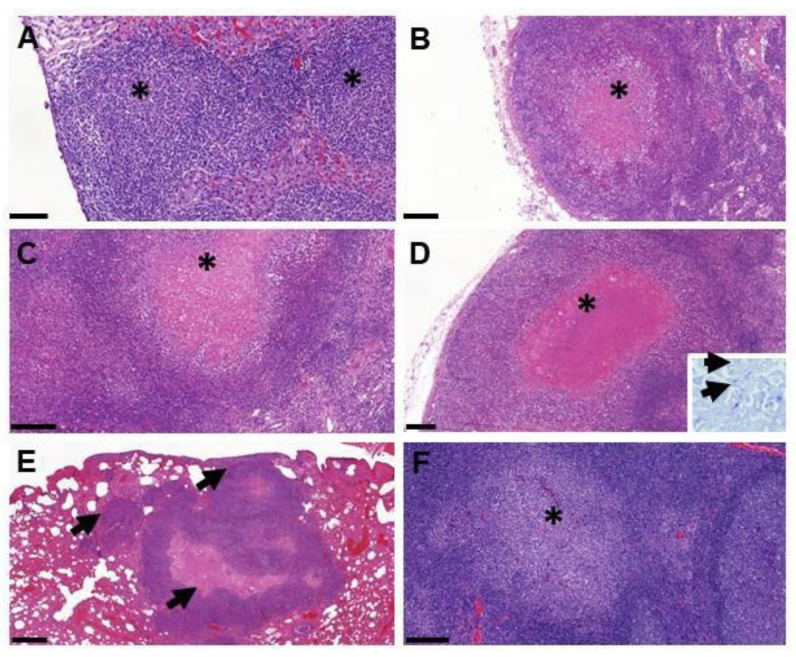
Examples of lesions observed by histopathology in foxes. (**A**) Multiple coalescing type I granulomas (*) within the liver (fox D); bar = 100 µm. (**B**) Necrotic type III granuloma (*) in the periphery of the mandibular lymph node (fox B); bar = 200 µm. (**C**) Necrotic type III granuloma (*) in the retropharyngeal lymph node; bar = 200 µm. (**D**) Large type IV granuloma with caseotic necrosis (*) in the mesenteric lymph node (fox G); bar = 200 µm; insert = acid-fast bacilli (arrows) within the necrotic area stained by the Ziehl–Neelsen technique. (**E**) Multiple type IV granulomas (arrows) within the lung parenchyma (fox I); bar = 500 µm. (**F**) Solid type I granuloma (*) in the tonsil (fox D).

TB granulomas were detected in at least 1 tissue from 8 out of the 12 infected animals (Table S2). The main tissues affected were the lymph nodes, mostly left and right mandibular and retropharyngeal lymph nodes, but also hepatic and mesenteric lymph nodes (Figure 5). Most of the lesions presented very few AFB, except in the mesenteric lymph node from one of the animals (fox G) (data not shown). Small early stage granulomas (type I–II) were observed in the tonsils of three animals (foxes D, J and L) but in absence of AFB. Larger and more developed granulomas with a necrotic core (type III–IV) were also observed in the head and neck lymph nodes of several foxes, in the mesenteric lymph nodes of fox G and the lungs from fox I. Granulomas with few AFB were also observed in the liver of one animal (fox D) and in the lungs (fox I).

## 4. Discussion

In our study, 12 foxes were successfully orally infected with a field *M. bovis* strain, all being diagnosed as positive for *M. bovis* by bacterial culture and/or PCR in at least 1 tissue. The rationale for the oral infectious route was based on data obtained in Portugal and France showing mainly intestinal TB in wild foxes, thus strongly suggesting oral infection of foxes in natural conditions [6,8,14]. Data in Spain also suggested that wild foxes became infected with TB by scavenging on infected wild ungulate carcasses [5]. In this experimental study, 12 foxes were orally challenged with a high *M. bovis* infectious dose of 2.10^7^ CFU in 1 ml, in line with other experimental *M. bovis* oral infection protocols in carnivores [14,15]. Such a dose is usually higher than for endobronchial routes (usually in the range of 10^3^–10^4^ CFU) [16], and is relevant given high bacterial levels that can be recovered in dead animal carcasses accessible to foxes for scavenging (figures based on the French Tuberculosis National Reference Laboratory, data not published). Since the anatomopathological patterns and anatomical localization of infection found in the present study are similar to those observed in the wild, and given that the same strain of *M. bovis* circulates in a multi-host system in these areas, it can be hypothesized that most foxes acquire infection by the oral route *in natura*.

All foxes except one were seropositive to MPB83 during the experiment, and nine at the final time point, suggesting a serological response quite comparable to that observed in badgers experimentally infected by the endobronchial route with lower *M. bovis* doses [17,18,19]. The serological platforms we used (the ELISA IDEXX *M. bovis* Ab assay and MAPIA) all showed recognition of infection markers in foxes. The five foxes detected by MAPIA in IgG response (foxes A, E, G, I and L) were also positive by IDEXX at 12 wpi and three of them (G, I and L) were among the most heavily-infected animals according to bacterial detection. This relationship between the severity of pathology and seropositivity are commonly observed in animal TB [20]. In experimental studies with *M. bovis*-infected badgers, animals with more severe lesions tended to excrete more bacteria [21,22]. One fox (G) was positive at T0 by the IDEXX test, i.e., *M. bovis* before infection, but we did not identify at post-mortem any other pathogen (such as *Mycobacterium microti* [17] investigated separately by PCR) that may have caused cross-reactions to MPB83 and MPB70 coated on IDEXX plates.

At post-mortem, no visible lesion was found in the infected animals. Moreover, the histopathological analysis showed only a small number of lesions in 8 out of 12 animals. The scoring of granuloma severity in H&E stained tissues was based on the same scale as for the other species, such as cattle, sheep and badgers [10,11,23,24]. In types I and II granulomas, bacteria tended to be less numerous than in types III and IV [25]. Type II granulomas were the most frequent in the experimentally-infected foxes, in a similar manner to what was found in sheep [26], and low levels of types III or IV, suggesting a slow progression of the disease. The few tissues exhibiting lesions were mostly the mandibular and retropharyngeal lymph nodes and the mesenteric lymph nodes, draining the oronasal cavity and the abdominal gastrointestinal tract, respectively, in accordance to the oral route of infection. The lesions were comparable to those observed in badgers, including the absence of multinucleated giant cells (MNGCs) or mineralization within the necrotic cores seen in other species, such as deer [27]. The fact that the severity of pathological lesions was not very advanced can be partly related to the short duration of the study, the dose (maybe too low to induce more severe lesions) or the route of infection (oral) used in this experiment. The French *M. bovis* strain used in this study has not been delivered in experimental infections in other species; therefore, a strain effect in pathogenicity cannot thus be excluded. However, such strain effect is considered unlikely given that lesions of naturally-infected badgers with this or any strain are similar, which confirms the general observation that carnivores develop mild lesions as a result of TB [28].

The most heavily-infected tissues were the retropharyngeal lymph nodes and tonsils, as reported in wild, naturally-infected ferrets [29], and the mesenteric lymph node as observed in TB-infected foxes in the Doñana Park [5]. In studies with wild boar and feral pigs [30,31], natural oral infection caused TB gross lesions in the tonsils and mandibular lymph nodes, although, in the wild boar, they were also found in lung and mesenteric lymph nodes. Tonsils, as observed in this study, are also one of the main tissues affected in infections in cattle and cervids, suggesting that tonsils should be analyzed together with mandibular, retropharyngeal, hepatic and mesenteric lymph nodes to diagnose TB in red foxes.

In this study, six foxes showed positive results by culture and/or PCR in the mesenteric lymph nodes, as well as in feces. However, only one exhibited a lesion in those lymph nodes. It can therefore be suggested that mycobacteria may be shed in feces when intestinal tissues are affected, even in absence of gross or microscopic lesions [31]. The highest positive results in feces were obtained a few weeks after infection. It cannot be fully excluded that the detected *M. bovis* belonged to the original inoculum still being cleared from the digestive track rather than following local bacterial replication. However, even larger oral doses (>10^9^ CFU) of attenuated *M. bovis* (BCG) appeared to be cleared from the digestive track and undetectable in badger feces within 48 h [32]. It seems unlikely that *M. bovis* would survive for weeks in the digestive track without inducing mucosal uptake and local infection, as seen with BCG within 8 weeks [13]. The gastric fluids are likely to inactivate *M. bovis*, therefore live *M. bovis* will probably only be detected by the culture from lesions located in the pre-gastric digestive track. Positive results obtained by PCR in the lower digestive tract can reflect the excretion of *M. bovis* but and also the presence of dead bacilli. Moreover, we cannot rule out that the lower level of *M. bovis* detection by bacteriology compared to PCR was not the consequence of freezing and thawing of feces that is particularly detrimental for mycobacterial survival, but not for molecular identification. Kidneys and urine samples were negative by culture and PCR, suggesting that this was not a major route of excretion, or may have occurred under a longer study duration. A previous field investigation considered the excretion of *M. bovis* in urine [6]. The oropharyngeal cavity appeared as infected (with potential excretion through saliva as in pigs [33]) since 3 animals presented early stage TB granulomas in their tonsils. Tonsils may have been an early site of *M. bovis* entry following oral inoculation, as seen for BCG in badgers [13]. Overall, 10 foxes were positive in tonsils by culture and/or PCR, and 2, 1 and 2 of them were positive in oropharyngeal swabs by PCR at 4, 8 and 12 wpi, respectively. Severe infection of the thoracic cavity was not observed in this study, but this may have occurred under a longer study duration.

## 5. Conclusions

All 12 foxes were successfully infected with a field *M. bovis* strain delivered orally, with *M. bovis* detected by culture or PCR in at least 1 tissue per animal. The majority of animals (11/12 foxes) exhibited active infection with *M. bovis*. The infection had no clinical impact within the 12-week study and the pathological lesions (either macroscopic or by histology) were mild. In 9, 2 and 3 foxes, *M. bovis* was detected in the feces, in the oropharyngeal mucus and in the bladder at post-mortem, respectively. Therefore, this original oral experimental infection was able to reproduce the pattern of infection observed in naturally TB-infected foxes in the wild and demonstrated a risk of mycobacteria shedding by this species. Future experimentation using lower infectious doses but with repetitive oral uptakes and during longer time courses, which might reflect another means of natural exposure of foxes to contaminated scavenge, could be assessed in order to gain further insight into our knowledge on the pathology of TB in foxes. Although our results have to be taken into account when studying the putative role of foxes in the multi-host epidemiological system for TB, they have to be nonetheless considered and interpreted together with the ecological traits of foxes. The combination of both will inform on the probability of intra- and interspecific contacts and thus on the ability of potential transmission. In summary, we reproduced TB infection in the fox and set up a useful infectious experimental model in this animal species for the eventual vaccination assays programmed as the future interventions for limiting the role of foxes in multi-host transmission systems in infected regions in France.

## Figures and Tables

**Table 1:** Characteristics of foxes experimentally infected with *Mycobacterium bovis* (sex and age), and the results of serological tests (ELISA IDEXX and MAPIA) and number of tissues positive in histology (granuloma), bacterial culture and molecular diagnostic (PCR). For MAPIA, antigens eliciting IgM and IgG antibody responses; wpi: weeks post infection.

Fox ID	Sex	Age (in Years)	ELISA Idexx				MAPIA		End-point (T3–12 wpi)		
			T0	T1	T2	T3	Antigens Recognized (T1–T3)		Tissues with		
				(4 wpi)	(8 wpi)	(12 wpi)	IgG Response	IgM Response	Granuloma	Culture Positive	PCR Positive
**A**	M	1	-	+	-	+	B-PPD	-	0	2	4
**B**	F	7	-	+	-	+	-	DID38, DID65	4	4	7
**C**	M	1	-	+	+	+	-	DID65	0	3	4
**D**	F	1	-	-	-	-	None		2	5	5
**E**	F	3	-	+	+	+	E6/P10, DID65, B-PPD	-	1	3	5
**F**	M	1	-	+	-	+	None		0	0	3
**G**	M	3	+	+	+	+	E6/P10, DID38, B-PPD	MPB83, DID38	2	6	7
**H**	F	1	-	+	+	-	None		2	3	4
**I**	F	7	-	+	+	+	E6/P10, DID38, DID65, B-PPD	-	3	6	7
**J**	M	3	-	-	-	-	-	B-PPD	1	3	6
**K**	F	3	-	+	-	+	-	DID65	0	1	4
**L**	M	3	-	+	-	+	MPB83, DID38, DID65	MPB83, E6/P10, DID38, DID65	3	4	5

**Table 2:** Results in bacteriology (Bac) (in blue) and PCR (IS*6110* and IS*1081*) (in yellow) of the different positive tissues tested in the 12 foxes experimentally infected with *Mycobacterium bovis* from the post-mortem analyses (positive tissue: tissue exhibited positive result(s) in bacteriology and/or PCR) in at least one fox. Abs: Absent tissue. (−) Inhibited tissue in IPC. 1: positive result in PCR. +: Positive in bacteriology. -: negative. L: left. R: right. LN: lymph node.

Type of Sample	A		B		C		D		E		F		G		H		I		J		K		L		Total	
	Bac	PCR	Bac	PCR	Bac	PCR	Bac	PCR	Bac	PCR	Bac	PCR	Bac	PCR	Bac	PCR	Bac	PCR	Bac	PCR	Bac	PCR	Bac	PCR	BAC	PCR
Tonsils	+	+	+	+	+	+	+	+	+	+	-	-	+	+	+	+	+	+	-	+	-	+	-	-	**8**	**10**
Right retropharyngeal LN	-	+	+	+	+	+	+	+	+	+	-	+	-	+	+	+	+	+	+	+	-	+	-	-	**7**	**11**
Left retropharyngeal LN	+	+	+	+	+	+	+	+	-	+	-	+	+	+	+	+	+	+	-	-	-	+	+	+	**8**	**11**
Right mandibular LN	-	-	-	+	-	-	+	+	-	+	-	-	+	+	-	-	+	+	-	+	-	-	-	-	**3**	**6**
Left mandibular LN	-	-	+	+	-	-	-	-	+	+	-	-	+	+	-	-	+	+	-	-	-	-	-	+	**4**	**5**
Left parotid LN	-	-	-	-	-	+	-	-	-	-	-	-	-	-	-	-	-	-	-	-	-	-	+	+	**1**	**2**
Heart	-	-	-	+	-	-	-	-	Abs	Abs	Abs	Abs	Abs	Abs	Abs	Abs	Abs	Abs	Abs	Abs	Abs	Abs	Abs	Abs	**0**	**1**
Hepatic LN	-	-	-	+	Abs	Abs	-	-	-	-	-	-	+	+	-	-	-	-	+	+	-	-	+	+	**3**	**4**
Liver	-	-	-	-	-	-	+	+	-	-	-	-	-	-	-	-	-	+	-	+	-	-	+	+	**2**	**4**
Mesenteric LN	-	+	-	-	-	-	-	-	-	-	-	+	+	+	-	-	+	+	+	+	+	+	-	-	**4**	**6**
Kidneys (L & R)	-	-	-	-	-	-	-	-	-	-	-	-	-	-	-	+	-	-	-	-	-	-	-	-	**0**	**1**
**Total**	**2**	**4**	**4**	**7**	**3**	**4**	**5**	**5**	**3**	**5**	**0**	**3**	**6**	**7**	**3**	**4**	**6**	**7**	**3**	**6**	**1**	**4**	**4**	**5**	**40**	**61**

## Data Availability

Not applicable.

## Supplementary Materials

The following supporting information can be downloaded at: https://www.mdpi.com/article/10.3390/microorganisms10020380/s1: Figure S1. IgG antibody responses to *M. bovis* antigens in experimentally-infected foxes at day 0 (T0), T1 (4 weeks post infection, wpi) and T3 (12 wpi). MAPIA was performed as described in the Materials and Methods Section. Animal identification numbers are shown to the left, whereas antigen names are shown to the right of each strip panel. Any visible band of greater intensity than that observed at week 0 (pre infection) is considered an antibody-positive result. Table S1. Amount of tissue processed for bacterial culture and molecular diagnosis per sample by fox. Table S2. Granulomas observed in each animal and organ. The number shows the highest granuloma stage (I to IV) observed in each tissue. * Acid-fast bacilli present in Ziehl–Neelsen stained sections. Table S3. Results of bacteriology and molecular diagnostic from tissue and shedding material for each of the twelve foxes. Abs: Absent tissue. indet: no *Mycobacterium bovis* DNA amplification. Neg: no bacterial colony in culture. Table S4. Results of bacteriology and molecular diagnostic on shedding material for each of the twelve foxes at the different time-points of the study. Abs: Absent tissue. indet: no *Mycobacterium bovis* DNA amplification. Neg: no bacterial colony in culture.
