# Supplementary figures and images for: Experimental Infection of Captive Red Foxes (Vulpes vulpes) with Mycobacterium bovis
Source: Microorganisms. 2022 Feb 6;10(2):380. doi: 10.3390/microorganisms10020380 (PMC8879846; doi:10.3390/microorganisms10020380)

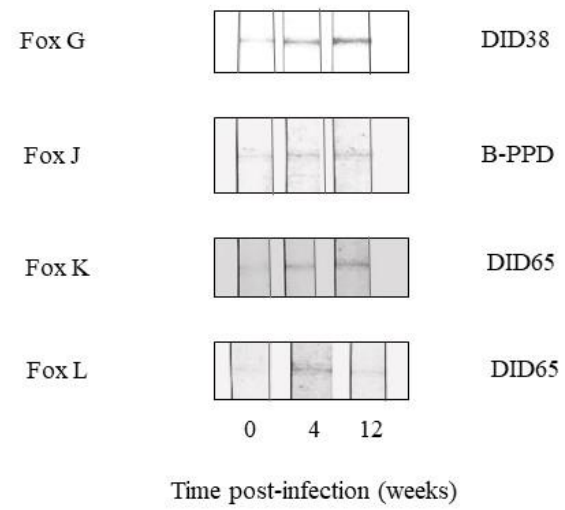

Supplementary figure

Supplement: Supplementary file 1 [file microorganisms-10-00380-s001.zip › supplementary/Supplementary figure.pdf]
